# Supplementary material for: Enhancers regulate 3′ end processing activity to control expression of alternative 3′UTR isoforms
Source: Nat Commun. 2022 May 17;13:2709. doi: 10.1038/s41467-022-30525-y (PMC9114392; doi:10.1038/s41467-022-30525-y)
Supplement: Supplementary file 1 — Supplementary information [file 41467_2022_30525_MOESM1_ESM.pdf]

Supplementary Information

Enhancers regulate 3′ end processing activity to control expression of alternative 3′UTR isoforms

Buki Kwon[1], Mervin M. Fansler[1,2], Neil D. Patel[1], Jihye Lee[1], Weirui Ma[1], and Christine Mayr[1,2†]

[1]Cancer Biology and Genetics Program, Memorial Sloan Kettering Cancer Center, New York, NY 10065, USA

[2]Tri-Institutional Training Program in Computational Biology and Medicine, Weill Cornell Graduate College, New York, 10021, NY, USA

[†]Correspondence: mayrc@mskcc.org

Supplementary Table 1. Sequences of PAS, promoters, and enhancers

A. PAS sequences

Gene	PAS type	Sequence
SV40	PAS	TTCGAGCAGACATGATAAGATACATTGATGAGTTTGGACAAACCACAACTAGAATGCAGTGAAAAAAATGCTTTATTTGTGAAATTTGTGATGCTATTGCTTTATTTGTAACCATTATAAGCTGCAATAAACAAGTTAACAACAACAATTGCATTCATTTTATGTTTCAGGTTCAGGGGGAGGTGTGGGAGGTTTTTTAAAGCAAGTAAAACCTCTACAAATGTGGTAAA
BGH	PAS	CTGTGCCTTCTAGTTGCCAGCCATCTGTTGTTTGCCCCTCCCCCGTGCCTTCCTTGACCCTGGAAGGTGCCACTCCCACTGTCCTTTCCTAATAAAATGAGGAAATTGCATCGCATTGTCTGAGTAGGTGTCATTCTATTCTGGGGGGTGGGGTGGGGCAGGACAGCAAGGGGGAGGATTGGGAAGACAATAGCAGGCATGCTGGGGATGCGGTGGGCTCTATG
PTEN	PPAS	ATTTTTTTTTATCAAGAGGGATAAAACACCATGAAAATAAACTTGAATAAACTGAAAATGGACCTTTTTTTTTTTAATGGCAAT
NUDT21	PPAS	GAAGTAAAAGAAGCCGCTTGTCTCTGTGAGCACAGCTATATACAGTGTAGAATAAATGTGGTAGAAAAGTTTTTTTGGTTTTATCTCTTTTGCGATCCCT
DICER1	PPAS	GAATTGAAGGCAGAATTTAAAGTTTGGTTGATAACAGGATAGATAACAGAATAAAACATTTAACATATGTATAAAATTTTGGAACTAATTGTAGTTTTAG
RALA	PPAS	TCAAAGCCCAAACTCCTTTCTTATCTTGACCATACTAATAAATATAATTTATAAGCATTGCCATTGAAGGCTTAATTGACTGAAATTACTTTAACATTTT
SPN (CD43)	PPAS	CGCCTGGGCAGGGCAGGGCTGGCACCTCTCAACGTCTGTGGACTGAATGAATAAACCCTCCTCATCCACCCCTATTTATCTCCATCACCATTTCCCCCTCTTTCTTGTTCC
CEBPG	PPAS	ATGTTAAGGTATATGGTAAAAAAAGAAATATCTGGGATCCCGATGTTCTTAATAAATCCTGACTTCCCAAGAAATGCTTCTTTTTTAAGTTGACAAAAGG
PTEN	DPAS	GTTTGTTAACAACCCTTTATCTCTTAGTGTTATAAACTCCACTTAAAACTGATTAAAGTCTCATTCTTGTCATTGTGTGGGTGTTTTATTAAATGAGAGTT
NUDT21	DPAS	AAGTTTGGTTAAAGTGATCACTGTTCTTTTTTTATTTTATTGTCATTTCAATAAAAAATATTTGAAAGAGAATGACTCTGTGATTGTTTTGGGATAGTA
GAPDH	PAS	CCGGAAGAGGGGAGGGGCCTAGGGAGCCGCACCTTGTCATGTACCATCAATAAAGTACCCTGTGCTCAACCAGTTACTTGTCCTGTCTTATTCTAGGG
UBC	PAS	GTTTCCCCTTTTAAGGTTTCAACAAATTTCATTGCACTTTCCTTTCAATAAAGTTGTTGCATTCCCAATTGGTGTTTTTTTTTTTTTTTTTTTTTAAGTTTATTTTGCATTAGACTGGG

B. Promoters

Promoter	Abbreviation	Refseq-ID	Start with respect to TSS	End with respect to TSS	Total length (bp)
PTEN	Pprom	NM_000314	-178	414	592
NUDT21	Nprom	NM_007006	-1,262	706	1,968
GAPDH	Gprom	NM_002046	-2,112	1,727	3,839
TATA	TATA	NA	caattgaattgtagagggtatataatggaagctcgacttccaga		44

C. Enhancers

Enhancer	Abbreviation	Refseq-ID of nearest gene	Start with respect to TSS	End with respect to TSS	Total length (bp)
PTEN	Penh	NM_000314	-2,272	-179	2,094
PTEN	Penh1	NM_000314	-1,185	-179	1,007
Distal	Denh	NM_001144	+53,649	+55,738	2,090

Supplementary Table 2. Primer sequences

**Deletion of PTEN enhancer using CRISPR-Cas9**	
gRNA1-F	CACCGAGTGTTCTTAGAGACCTCTG
gRNA1-R	AAACCAGAGGTCTCTAAGAACACT
gRNA2-F	CACCGCAGAAACCCAGCCGGAGGCA
gRNA2-R	AAACTGCCTCCGGCTGGGTTTCTG
**Northern blotting**	
PTEN-NB-F	GCACAAGAGGCCCTAGATTT
PTEN-NB-R	TCTCTGGATCAGAGTCAGTGGT
GAPDH-NB-F	ACAACTTTGGTATCGTGGAAGG
GAPDH-NB-R	TTACTCCTTGGAGGCCATG
**qPCR**	
Outside of PTEN enhancer (#1)-F	CTCAGGAGCAACCAACTCAACC
Outside of PTEN enhancer (#1)-R	TGTCCCAAATCTCTCCTGAAGC
Outside of PTEN enhancer (#2)-F	CCATGGAATCCAAGAGGATTCT
Outside of PTEN enhancer (#2)-R	GCCTACCCTAAGTGACAATTAG
Inside of PTEN enhancer (#3)-F	TGCGATCCAACTCTCAGCATTTCC
Inside of PTEN enhancer (#3)-R	TGAGAACCTAGTCAATGGCCGTCA
Inside of PTEN enhancer (#4)-F	CCTCAAGCACAGAACCAAAAG
Inside of PTEN enhancer (#4)-R	CTCACTCCTTGCTCTACATCG
PTEN-qP-F	AAGGGACGAACTGGTGTAATG
PTEN-qP-R	GCCTCTGACTGGGAATAGTTAC
RPL19-qP-F	AAAACAAGCGGATTCTCATGGA
RPL19-qP-R	TGCGTGCTTCCTTGGTCTTAG
PTEN-int1-qP-F	TCGGTAATCCGGTCTCCTAA
PTEN-int1-qP-R	GATGCAATATGCGTACGTGC
yACT1-qP-F	TGGATTCCGGTGATGGTGTT
yACT1-qP-F	ACCGGCCAAATCGATTCTCA
RenORF-qP-F	TTGTGCCACATATTGAGCCA
RenORF-qP-R	TAAGAAGTTCAAACCATGCAG
RenRT-qP-F	GGATGAACGAAATAGACAGATCG
RenRT-qP-R	AGGATCTTCACCTAGATCCT
PTEN-stability-F	GTCCAGAGCCATTTCCATCCT
PTEN-stability-R	TCTTTCTGCAGGAAATCCCAT
Rluc-stability-F	TGAGCATCAAGATAAGATCAAAGC
Rluc-stability-R	AGATATGCTGCAAATTCTTCTGG
GAPDH-stability-F	GCGAGATCCCTCCAAAATCAA
GAPDH-stability-R	GTTCACACCCATGACGAACAT
PTENLU-qP-F	TGTGCAGTGTTGAATCATTTCTTC
PTENLU-qP-R	CTTCACATTAGCTTTACAATAGTAG
Ren-Rolling-F	TCAAGAGCTACCAACTCTTTT
Ren-Rolling-R	AAAGATACCAGGCGTTTCCC
RenORF-Rolling-R	TCATCACTTGCACGTAGATAAGC
**Determination of splicing patterns of the endogenous PTEN gene**	
PTEN-sp-F	ATGACAGCCATCATCAAAGAGATC
PTEN-sp-R	TCAGACTTTTGTAATTTGTGTATGC
**5′RACE**	

PTEN-5'RACE-R	TGATACTTACCTGCCCAGTGC
PTEN-5'RACE-R2	GGTTAGAAAAGACGAAGAGGAGG

Kwon et al., Supplementary Fig. 1

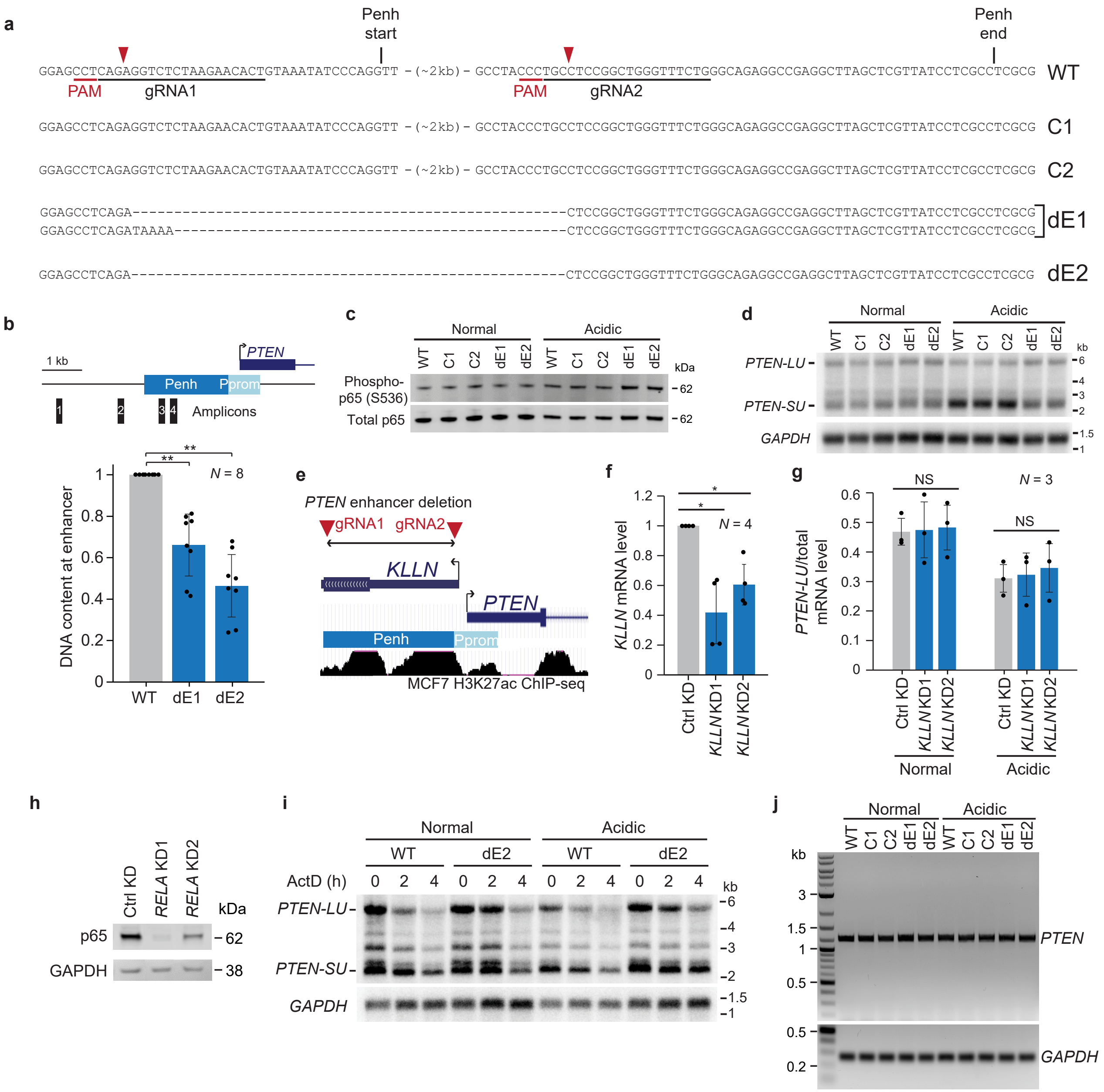

Supplementary Figure 1. CRISPR-mediated heterozygous deletion of the *PTEN* enhancer.

(a) Sequence alignment of *PTEN* alleles spanning at the boundaries of the PTEN enhancer deletion in the indicated samples. The predicted cutting sites of the gRNAs are marked by red arrow heads.

(b) Schematic showing strategy to assess the number of deleted alleles upon CRISPR-mediated *PTEN* enhancer deletion. As MCF7 cells contain more than two alleles for chromosome 10, four PCR amplicons were designed and the DNA content within the deletion was compared to the outside region and normalized to the WT sample. Shown is the fold change in DNA content in the *PTEN* enhancer region in dE1 and dE2 clones compared with WT cells. Data are shown as mean ± std of n = 8 biologically independent experiments. One-way ANOVA with Tukey's post-hoc test was performed. **, P = 0.001.

(c) Representative western blot showing steady-state protein levels of phosphorylated p65 (S536) and total p65 in the indicated samples. *n* = 3 biologically independent experiments.

(d) Representative northern blot of *PTEN* transcripts in the indicated samples. As in Fig. 1f but including two clonal WT cells (C1 and C2). *GAPDH* serves as loading control. *n* = 3 biologically independent experiments.

(e) UCSC genome browser snapshot showing the *PTEN* and *KLLN* gene loci with corresponding ChIP-seq data. CRISPR-mediated deletion of Penh by the two guide RNAs (red arrow heads) also deletes the *KLLN* gene.

(f) Steady-state levels of *KLLN* mRNA were measured by RT-qPCR in cells stably expressing the indicated shRNAs. Data are shown as mean ± std of *n* = 4 biologically independent experiments after normalization to *RPL19*. Two-tailed t-test for independent samples was performed. *, *P* =0.0029 between Ctrl KD and *KLLN* KD1 and *P* = 0.0024 between Ctrl KD and *KLLN* KD2..

(g) Steady-state levels of *PTEN-LU* and total *PTEN* mRNA were measured by RT-qPCR in the indicated samples. The fraction of *PTEN-LU* over total *PTEN* mRNA is shown as mean ± std of *n* = 3 biologically independent experiments. One-way ANOVA was performed. NS, not significant.

(h) Representative western blot showing steady-state levels of p65 protein in cells stably expressing the indicated shRNAs. GAPDH serves as loading control. *n* = 3 biologically independent experiments.

(i) Northern blot to determine the stability of *PTEN-SU* and *PTEN-LU* transcripts in WT and dE2 cells cultivated in normal or acidic conditions after actinomycin D (ActD) treatment for the indicated time points. *GAPDH* serves as the loading control. *n* = 4 biologically independent experiments.

(j) RT-PCR for full-length *PTEN* mRNA in the indicated samples. The *PTEN* mRNA from the start to the stop codon was amplified. *GAPDH* serves as loading control. *n* = 3 biologically independent experiments.
Source data for figures b-d and f-j are provided as a Source Data file.

Kwon et al., Supplementary Fig. 2

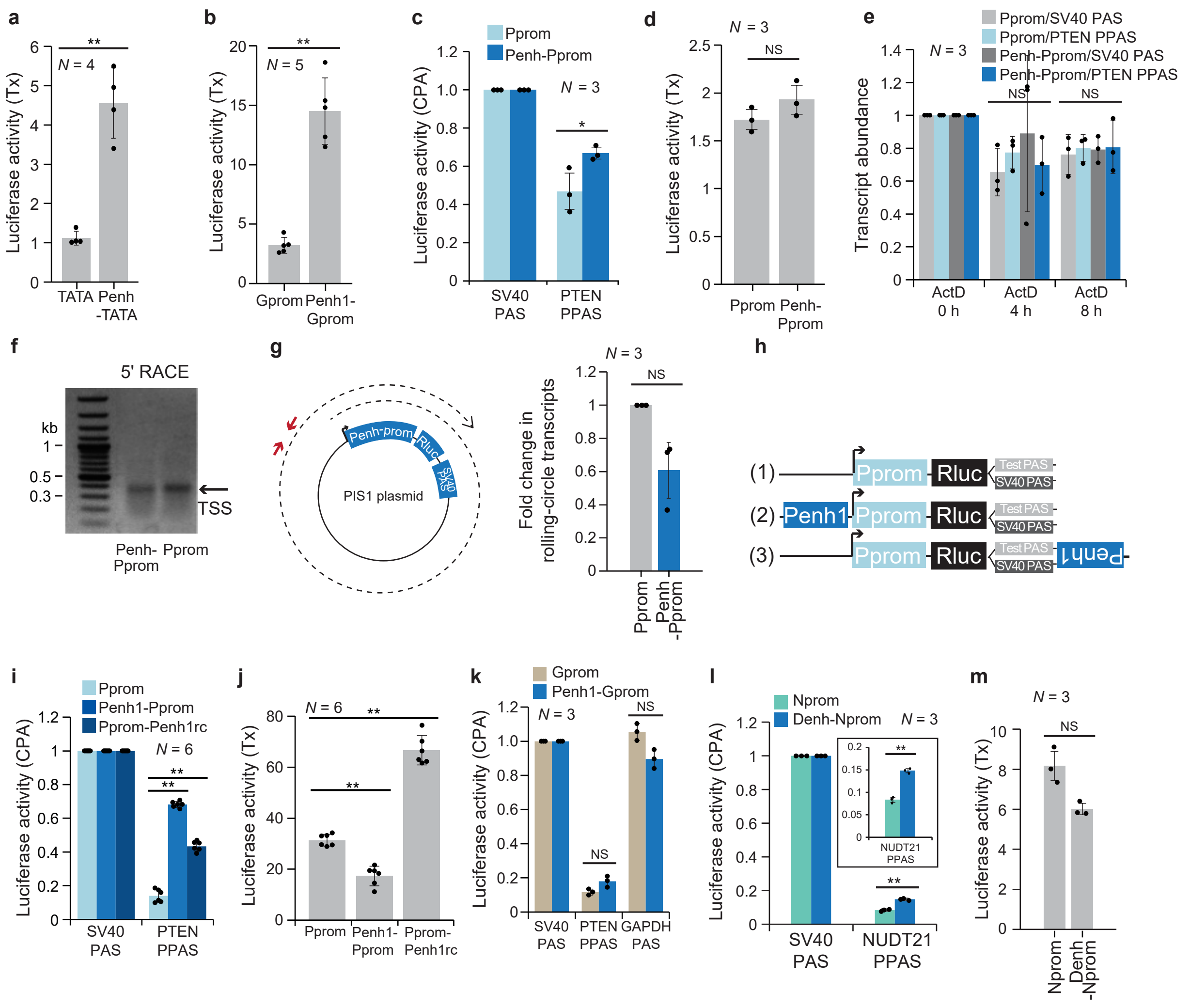

Supplementary Figure 2. Regulation of CPA activity by the *PTEN* enhancer.

(a) Luciferase activity corresponding to transcriptional activity (Tx) of a minimal synthetic promoter (TATA) was measured in the absence or presence of the *PTEN* enhancer (Penh-TATA) and is shown as in Fig. 2b. Data are shown as mean ± std of n = 4 biologically independent experiments. Two-tailed t-test for independent samples was performed. **, $P = 2 \times 10^{-4}$.

(b) Luciferase activity corresponding to transcriptional activity (Tx) of the *GAPDH* promoter (Gprom) in the presence or absence of the Penh1 shown as in Fig. 2b. Data are shown as mean ± std of n = 5 biologically independent experiments. Two-tailed t-test for independent samples was performed. **, $P = 5 \times 10^{-4}$.

(c) Relative CPA activity of the PPAS of PTEN when transcribed from the *PTEN* promoter in the absence or presence of the *PTEN* enhancer, after integration of the reporters into the genome of MCF7 cells using the Flp-in system. Data are shown as mean ± std of n = 3 biologically independent experiments. Two-tailed t-test for independent samples was performed. *, P = 0.048.

(d) Luciferase activity corresponding to transcriptional activity (Tx) of the Pprom reporter in the presence (Penh-Pprom) or absence (Pprom) of the *PTEN* enhancer after integration of the reporters into the genome of MCF7 cells using the Flp-in system, shown as in Fig. 2b. Data are shown as mean ± std of n = 3 biologically independent experiments. Two-tailed t-test for independent samples was performed. NS, not significant.

(e) Reporter mRNA transcript stability was assessed after actinomycin D (ActD) treatment of MCF7 cells expressing the indicated reporters at the indicated time points. The Penh did not influence mRNA stability of the reporters. The sequence context of the SV40 PAS and the PTEN PPAS did not influence the stability of the reporters. Data are shown as mean ± std of n = 3 biologically independent experiments. One-way ANOVA was performed. NS, not significant.

(f) 5′ RACE was used to determine the transcription start sites of the Pprom reporters in the presence or absence of the *PTEN* enhancer. The canonical transcription start site (TSS) is used in both reporters and is indicated by the arrow. n = 2 biologically independent experiments.

(g) Schematic of luciferase reporter constructs with possible rolling-circle transcription (dashed line). The primer pair (red arrows) at the intervening region of the plasmid was used to measure the levels of rolling-circle transcription by RT-PCR. Data are shown as mean ± std of n = 3 biologically independent experiments. Two-tailed t-test for independent samples was performed. NS, not significant.

(h) Schematic of luciferase reporter constructs to investigate position-dependent enhancer-mediated CPA cleavage activity. The reverse complement (rc) of Penh1 was cloned downstream of the PAS. Shown as in Fig. 2a.

(i) Luciferase activity corresponding to CPA activity of the PTEN PPAS measured using the reporter constructs shown in (h). Data are shown as mean ± std of n = 6 biologically independent experiments. Two-tailed t-test for independent samples was performed; **, $P = 1 \times 10^{-5}$.

(j) Luciferase activity corresponding to transcriptional activity (Tx) of the reporters from (h) shown as in Fig. 2b. Data are shown as mean ± std of n = 6 biologically independent experiments. Two-tailed t-test for independent samples was performed; **, $P = 1 \times 10^{-5}$.

(k) Luciferase activity corresponding to CPA activity in the context of the Gprom in the presence or absence of the Penh, shown as in Fig. 2c. Data are shown as mean ± std of n = 3 biologically independent experiments. Two-tailed t-test for independent samples was performed. NS, not significant.

(l) Luciferase activity corresponding to enhancer-dependent CPA activity in the context of the Nprom in the presence or absence of Denh. The cleavage activity of the NUDT21 PPAS was enlarged for clarity. Data are shown as mean ± std of n = 3 biologically independent experiments. Two-tailed t-test for independent samples was performed. **, P = 0.0002.

(m) Luciferase activity corresponding to transcriptional activity (Tx) of the reporters from (l) shown as in Fig. 2b. Data are shown as mean ± std of n = 3 biologically independent experiments. Two-tailed t-test for independent samples was performed. NS, not significant. Source data for figures a-e, g, and i-m are provided as a Source Data file.

Kwon et al., Supplementary Fig. 3

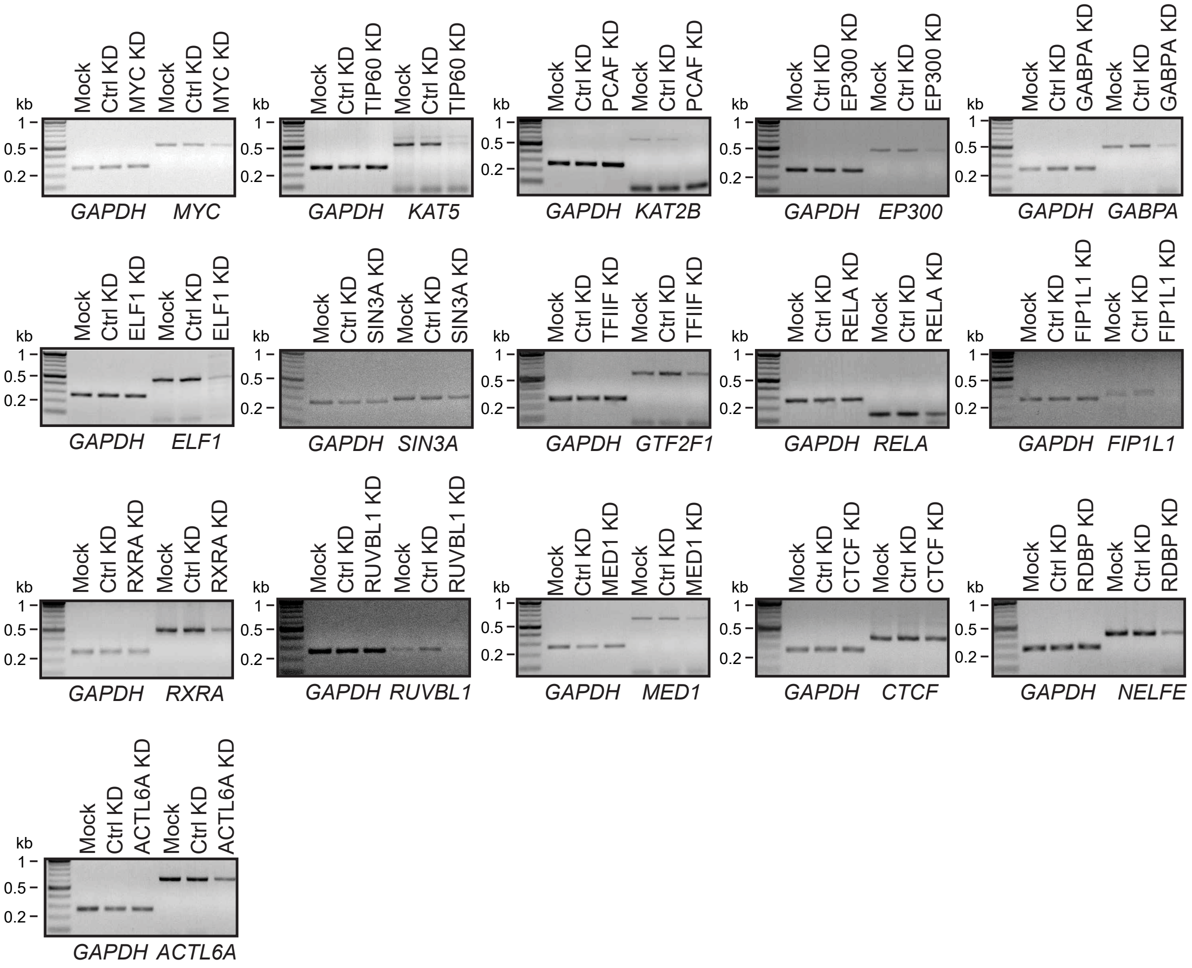

Supplementary Figure 3. shRNA-mediated knock-down of transcription factors and co-activators in MCF7 cells. Shown are mRNA levels of transcription factors and co-activators in MCF7 cells, after stable expression of a control shRNA (ctrl KD) or an shRNA against the indicated factor. Shown are stable cell lines which were generated once for each shRNA (*n* = 1). Mock, no shRNA was transfected. *GAPDH* serves as loading control. Source data are provided as a Source Data file.

Kwon et al., Supplementary Fig. 4

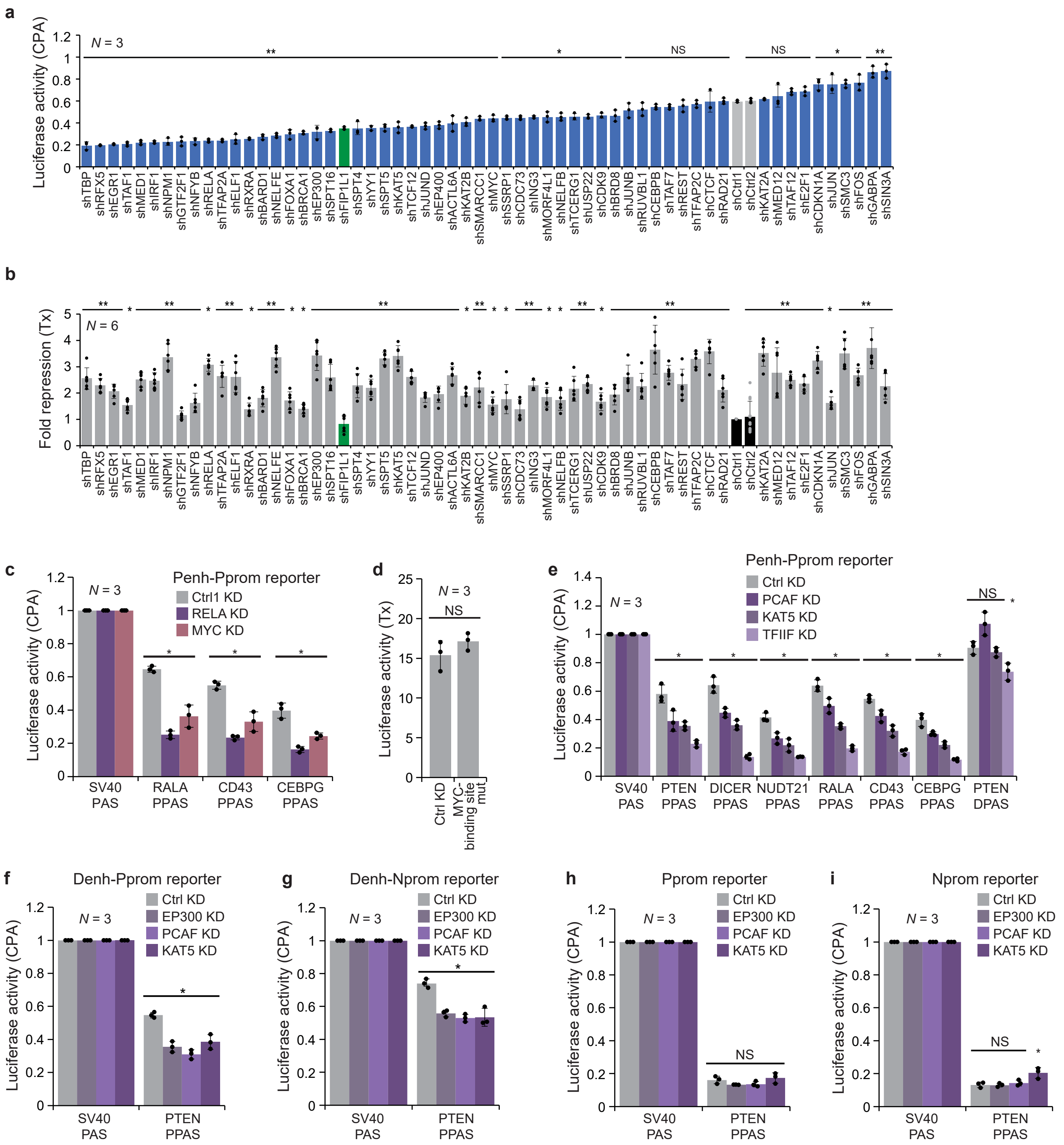

Supplementary Figure 4. shRNA screen identifies transcription factors and co-activators that regulate CPA activity of the PTEN PPAS in the context of the *PTEN* enhancer.

(a) Luciferase activity corresponding to CPA activity of the PTEN PPAS in the context of the Penh-Pprom reporter after KD of the indicated transcription factors or co-activators is shown as mean ± std. KD of FIP1L1 (green; CPA factor) serves as positive control. Data are shown as mean ± std of $n = 3$ biologically independent experiments. Two-tailed t-test for independent samples was performed; **, $P < 0.001$; *, $P < 0.02$. See Table 1 for values.

(b) Fold repression in transcriptional activity of the Penh-Pprom obtained after KD of the indicated transcription factors or co-activators relative to ctrl KD1. Data are shown as mean ± std of $n = 6$ biologically independent experiments. Two-tailed t-test for independent samples was performed. *, $P < 0.05$; **, $P < 0.0002$.

(c) Luciferase activity corresponding to CPA activity of additional PAS after KD of the indicated transcription factors. Data are shown as mean ± std of n = 3 biologically independent experiments. Two-tailed t-test for independent samples was performed. *, $P < 0.01$.

(d) Luciferase activity corresponding to transcriptional activity (Tx) of the Penh-Pprom reporter upon Ctrl KD or after mutation of the MYC-binding sites in the PTEN enhancer shown as in Fig. 2b. Data are shown as mean ± std of n = 3 biologically independent experiments. Two-tailed t-test for independent samples was performed. NS, not significant.

(e) Luciferase activity corresponding to CPA activity of additional PAS after KD of co-activators. Data are shown as mean ± std of n = 3 biologically independent experiments. Two-tailed t-test for independent samples was performed. *, $P < 0.02$.

(f-i) Luciferase activity corresponding to CPA activity of the PTEN PPAS in the context of different promoters in the presence or absence of the distal enhancer after of KD of histone acetyltransferases. Data are shown as mean ± std of n = 3 biologically independent experiments. Two-tailed t-test for independent samples was performed. **, $P < 0.001$; *, $P < 0.03$.
Source data for all the figures are provided as a Source Data file.

Kwon et al., Supplementary Fig. 5

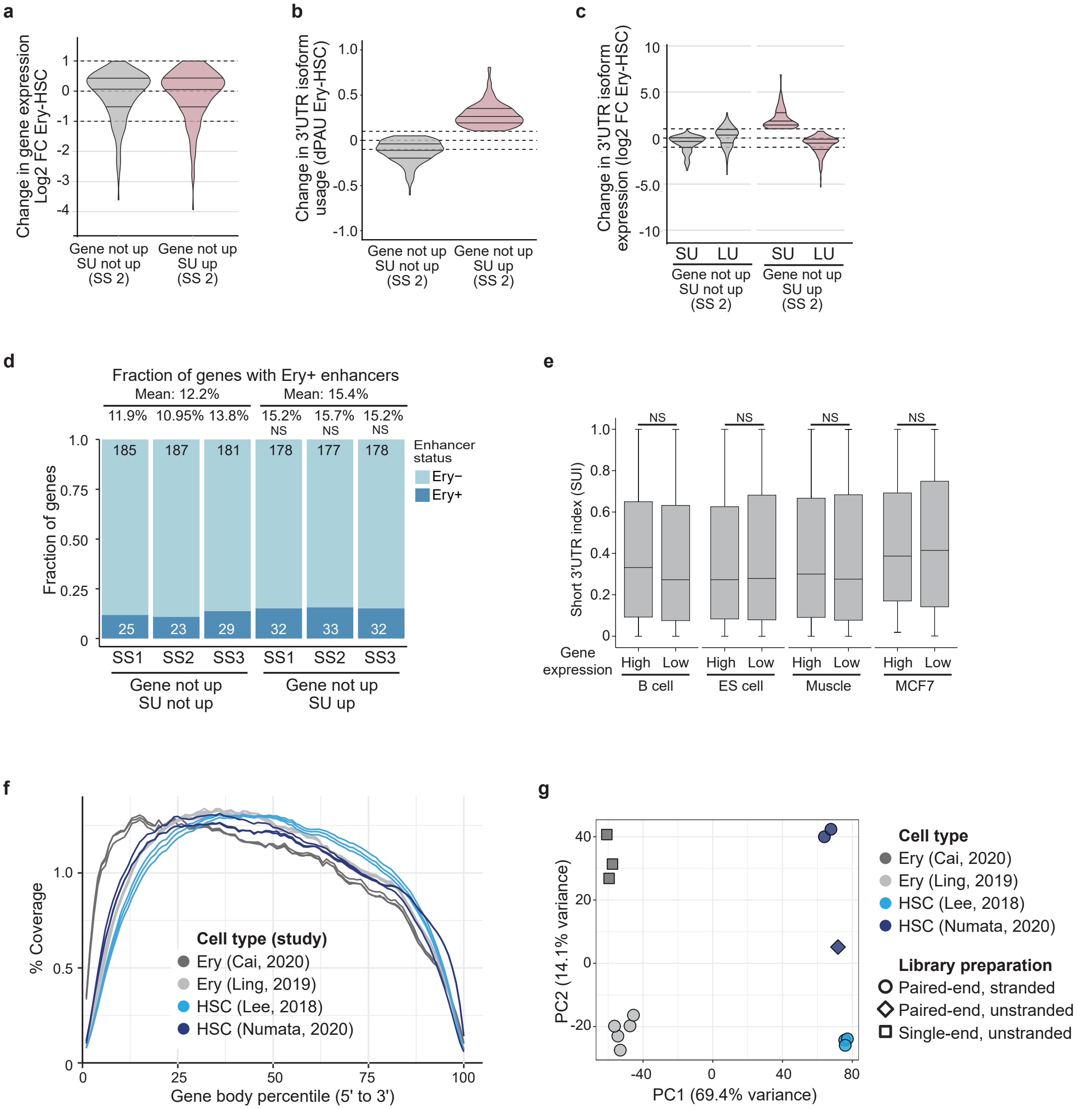

Supplementary Figure 5. Controlling for gene expression changes in genes with upregulation of SU isoforms.

(a) Stratified random sampling was used to fully control for gene expression changes between the ‘SU up’ group and the control group (which represents a subsample of the ‘gene not up’ group). For the control group, only genes without any upregulation of their *SU* isoforms were included (TPM FC in *SU* isoform between Ery vs HSC < 1.25-fold and dPAU < 0.05). Shown is the FC in gene expression in the indicated groups (control, *N* = 210, SU up, *N* = 210) for subsample 2 (SS2).

(b) Change in 3′UTR isoform ratio as determined by a change in PAU for the groups from (a).

(c) FC in 3′UTR isoform expression shown for *SU* and *LU* isoforms in the groups from (a).

(d) Fraction of genes associated erythroblast-specific enhancers (Ery+) in the groups from (a). Three random subsamples (SS) are shown for the control and the 'SU up' groups. Chi-square test: NS, not significant.

(e) The fraction of *SU* isoform expression of the total 3′UTR isoform expression (corresponding to the short 3′UTR index)[3] is shown for the 10% highest ($N \approx 400$) and 10% lowest ($N \approx 400$) expressed multi-UTR genes in the indicated cell types. Box plots show center line as median, box bounds as upper and lower quartiles, and whiskers as minimum to maximum values. Two-sided Mann-Whitney U-test was performed. NS, not significant.

(f) RNA-seq gene body coverage of four datasets on murine erythroblasts (Ery) and hematopoietic stem cells (HSC).

(g) Principal component analysis on the datasets from (a). In addition to variation caused by cell type-specific differences in gene expression (PC1), two of the datasets show large technical variations likely caused by different library preparation methods (PC2). The datasets with the least difference in technical variation (Lee, 2018 and Ling, 2019) were used for the analysis in Fig. 5.
