# Supplementary material for: Enhancers regulate 3′ end processing activity to control expression of alternative 3′UTR isoforms
Source: Nat Commun. 2022 May 17;13:2709. doi: 10.1038/s41467-022-30525-y (PMC9114392; doi:10.1038/s41467-022-30525-y)
Supplement: Supplementary file 3 — Reporting Summary [file 41467_2022_30525_MOESM3_ESM.pdf]

nature portfolio

Corresponding author(s): Christine Mayr

Last updated by author(s): Apr 20, 2022

Reporting Summary

Nature Portfolio wishes to improve the reproducibility of the work that we publish. This form provides structure for consistency and transparency in reporting. For further information on Nature Portfolio policies, see our Editorial Policies and the Editorial Policy Checklist.

Statistics

For all statistical analyses, confirm that the following items are present in the figure legend, table legend, main text, or Methods section.

n/a	Confirmed	
☐	☒	The exact sample size (*n*) for each experimental group/condition, given as a discrete number and unit of measurement
☐	☒	A statement on whether measurements were taken from distinct samples or whether the same sample was measured repeatedly
☐	☒	The statistical test(s) used AND whether they are one- or two-sided<br>*Only common tests should be described solely by name; describe more complex techniques in the Methods section.*
☒	☐	A description of all covariates tested
☒	☐	A description of any assumptions or corrections, such as tests of normality and adjustment for multiple comparisons
☐	☒	A full description of the statistical parameters including central tendency (e.g. means) or other basic estimates (e.g. regression coefficient) AND variation (e.g. standard deviation) or associated estimates of uncertainty (e.g. confidence intervals)
☐	☒	For null hypothesis testing, the test statistic (e.g. *F*, *t*, *r*) with confidence intervals, effect sizes, degrees of freedom and *P* value noted<br>*Give P values as exact values whenever suitable.*
☒	☐	For Bayesian analysis, information on the choice of priors and Markov chain Monte Carlo settings
☒	☐	For hierarchical and complex designs, identification of the appropriate level for tests and full reporting of outcomes
☐	☒	Estimates of effect sizes (e.g. Cohen's *d*, Pearson's *r*), indicating how they were calculated

Our web collection on statistics for biologists contains articles on many of the points above.

Software and code

Policy information about availability of computer code

Data collection	No software was used for data collection.
Data analysis	RNA-seq: Genomic alignment with HISAT2 v2.1.0. Gene body coverage was performed using RSeQC v4.0.0. MultiQC v1.10.1 was used to compare coverages. Gene expression differences were identified by DESeq2 v1.28. Differences in 3′UTR isoform expression were determined using QAPA v1.3.0 mm10. Pseudoalignment of samples using Salmon. Statistically significant changes in 3′UTR isoform usage were assessed using DEXSeq v1.34.0. All analysis was performed in R v4.0.2 and Bioconductor v1.13.<br>3′-seq: Obtained from SRP029953 (Lianoglou, 2013)<br>Western blot: Odyssey CLx imaging system v1.0.0.55 (Li-Cor).<br>Northern blot: Fuji phosphorimager v1.1.<br>Luciferase assay: Glomax 96 microplate luminometer and Glomax 96 software v1.9.2 (Promega)<br>Statistics: Excel v2203 (MS Office 16), R v4.0.2, and SPSS v.28.0.1.<br>Our code is available at https://github.com/Mayrlab/utr-enhancers

For manuscripts utilizing custom algorithms or software that are central to the research but not yet described in published literature, software must be made available to editors and reviewers. We strongly encourage code deposition in a community repository (e.g. GitHub). See the Nature Portfolio guidelines for submitting code & software for further information.

Data

Policy information about availability of data

All manuscripts must include a data availability statement. This statement should provide the following information, where applicable:

- Accession codes, unique identifiers, or web links for publicly available datasets
- A description of any restrictions on data availability
- For clinical datasets or third party data, please ensure that the statement adheres to our policy

We analyzed publicly available datasets. To identify the enhancers and promoters used in this study, levels of acetylated H3K27 and transcription factor binding sites in MCF7 cells were visualized using published ChIP-seq data (GSM946850), generated by the Encode project. Binding of MYC to the PTEN promoter was assessed by using published ChIP-seq data (GSE33213). FASTQ files for bulk RNA-seq samples were obtained from the Sequence Read Archive. Mouse definitive erythroblasts: SRR6946157-9, SRR8945139-41,44-45, mouse hematopoietic stem cells: SRR7946616-7, SRR6458998-9000. 3'-seq data were obtained from SRP029953.
Source data are provided with this paper.

Field-specific reporting

Please select the one below that is the best fit for your research. If you are not sure, read the appropriate sections before making your selection.

- [x] Life sciences
- [ ] Behavioural & social sciences
- [ ] Ecological, evolutionary & environmental sciences

For a reference copy of the document with all sections, see nature.com/documents/nr-reporting-summary-flat.pdf

Life sciences study design

All studies must disclose on these points even when the disclosure is negative.

Sample size	No sample size calculations were performed. The sample sizes were determined by the number of biological replicates required for ensuring statistical significance and chosen to support meaningful conclusions. The number of biological replicates is reported in the relevant figure legends in the manuscript.
Data exclusions	Due to a high technical variation in two of the RNA-seq datasets, we excluded them. See Extended Data Fig. 5 and methods for details.
Replication	For each experiment, at least triplicates were conducted with similar trends observed. The exact number of biologically independent samples is reported in each figure legend.
Randomization	Randomization is not relevant to this study since no in-vivo study was involved.
Blinding	Blinding to group allocation was not necessary since no group allocation was performed in this study.

Reporting for specific materials, systems and methods

We require information from authors about some types of materials, experimental systems and methods used in many studies. Here, indicate whether each material, system or method listed is relevant to your study. If you are not sure if a list item applies to your research, read the appropriate section before selecting a response.

Materials & experimental systems

n/a	Involved in the study	
☐	☒	Antibodies
☐	☒	Eukaryotic cell lines
☒	☐	Palaeontology and archaeology
☒	☐	Animals and other organisms
☒	☐	Human research participants
☒	☐	Clinical data
☒	☐	Dual use research of concern

Methods

n/a	Involved in the study	
☒	☐	ChIP-seq
☒	☐	Flow cytometry
☒	☐	MRI-based neuroimaging

Antibodies

Antibodies used	anti-PTEN (A2B1, Santa Cruz Biotechnology, sc-7974, 1:1000), anti-GAPDH (V-18, Santa Cruz Biotechnology, sc-20357, 1:500), anti-P65 (L8F6, Cell Signaling Technology, 6956, 1:1000), and anti-phospho-P65 Ser536 (93H1, Cell Signaling Technology, 3033, 1:1000), anti-mouse IRDye 800 (1:5000; Li-Cor Biosciences, Cat# 926-68072), anti-rabbit IRDye 680 (1:5000; Li-Cor Biosciences, Cat# 926–6807), and anti-goat IRDye 680 (1:5000; Li-Cor Biosciences, Cat# 926-32224)
Validation	All the antibodies used in this study are commercially available and validated by the manufacturers. All the primary antibodies were

Validation	validated to be suitable for western blot in human cell lines, as shown in the validation statements of the manufacturers' website.<br>anti-PTEN (A2B1, Santa Cruz Biotechnology, sc-7974, https://www.scbt.com/p/pten-antibody-a2b1)<br>anti-GAPDH (V-18, Santa Cruz Biotechnology, sc-20357, https://www.scbt.com/p/gapdh-antibody-v-18)<br>anti-P65 (L8F6, Cell Signaling Technology, 6956, https://www.cellsignal.com/products/primary-antibodies/nf-kb-p65-l8f6-mouse-mab/6956)<br>anti-phospho-P65 Ser536 (93H1, Cell Signaling Technology, 3033, https://www.cellsignal.com/products/primary-antibodies/phospho-nf-kb-p65-ser536-93h1-rabbit-mab/3033)

Eukaryotic cell lines

Policy information about cell lines

Cell line source(s)	MCF7 from the laboratory of Robert Weinberg (Whitehead Institute, Cambridge, USA), MCF7/FRT from the laboratory of Reuven Agami (Netherlands Cancer Institute, Amsterdam, Netherlands). The original commercial source of MCF7 is ATCC (HTB-22).
Authentication	We did not authenticate the cell line.
Mycoplasma contamination	All cell lines tested negative for mycoplasma contamination.
Commonly misidentified lines<br>(See ICLAC register)	No commonly misidentified cell lines were used in this study.
